# Wrist Joint Reconstruction With a Vascularized Fibula Free Flap Following Giant Cell Tumor Excision in the Distal Radius

**Published:** 2010-05-22

**Authors:** Chester J. Mays, Kyle Ver Steeg, Saeed Chowdhry, David Seligson, Bradon J. Wilhelmi

**Affiliations:** ^a^University of Louisville, Louisville, KY; ^b^Mount Sinai Medical Center, Chicago, Ill

## Abstract

**Objective:** Multiple therapeutic modalities exist for giant cell tumors (GCT) in the distal radius. The majority of GCTs are amenable to curettage, with the expanded lesions requiring a more radical approach. This case report examines the technique of managing a GCT that has extended beyond the boundaries of the cortex and into local tissues. The decision to use arthroplasty versus arthrodesis and the proximal fibular head as a vascularized free flap is discussed in reference to a patient requiring a proximal row carpectomy (PRC) secondary to tumor invasion. **Methods:** A 47-year-old woman with GCT in the right distal radius presented with decreased range of motion secondary to pain. Confirmation of the GCT was made with radiographic imaging and biopsy. The extensive invasion of the lesion required en bloc tumor resection with PRC and subsequent arthroplasty. **Results:** Treatment involved resection of tumor and PRC with arthroplasty using the proximal head of the fibula and reattachment of the radioscaphocapitate and ulnar carpal ligaments. Success was measured on functionality of the joint, viability of the flap, and the absence of tumor recurrence and pain. **Conclusion:** This case presents an example of successful excision of a GCT in the distal radius with a PRC and arthroplasty using a vascularized fibula free flap autograft. The patient remained pain-free, had no evidence of tumor recurrence, demonstrated 50% range of motion in the wrist, and 80% preoperative strength as expected following PRC.

Giant cell tumors (GCT) of the bone are rare, aggressive, benign neoplasms that account for 4% to 5% of primary bone tumors and 18.2% of benign bone tumors. Even though most are benign, 5% to 10% of patients may have a malignant, osteolytic lesion. The majority of cases are found in young adults between the ages of 20 and 40 years and are rarely seen in children or adults older than 65 years. The lesions are named for their histological appearance on microscopy showing many multinucleated “giant cells”. The etiology is usually spontaneous, as they are not known to be associated with trauma, environmental factors, or diet.^1^

## CASE PRESENTATION

A 47-year-old woman presented with restriction of movement in the right wrist because of an increase in pain. On examination she had tenderness of the right wrist over the distal radius and proximal carpal region. Active and passive motion was limited secondary to pain. The overlying skin was unremarkable, with no evidence of recent trauma.

**Figure 1:**
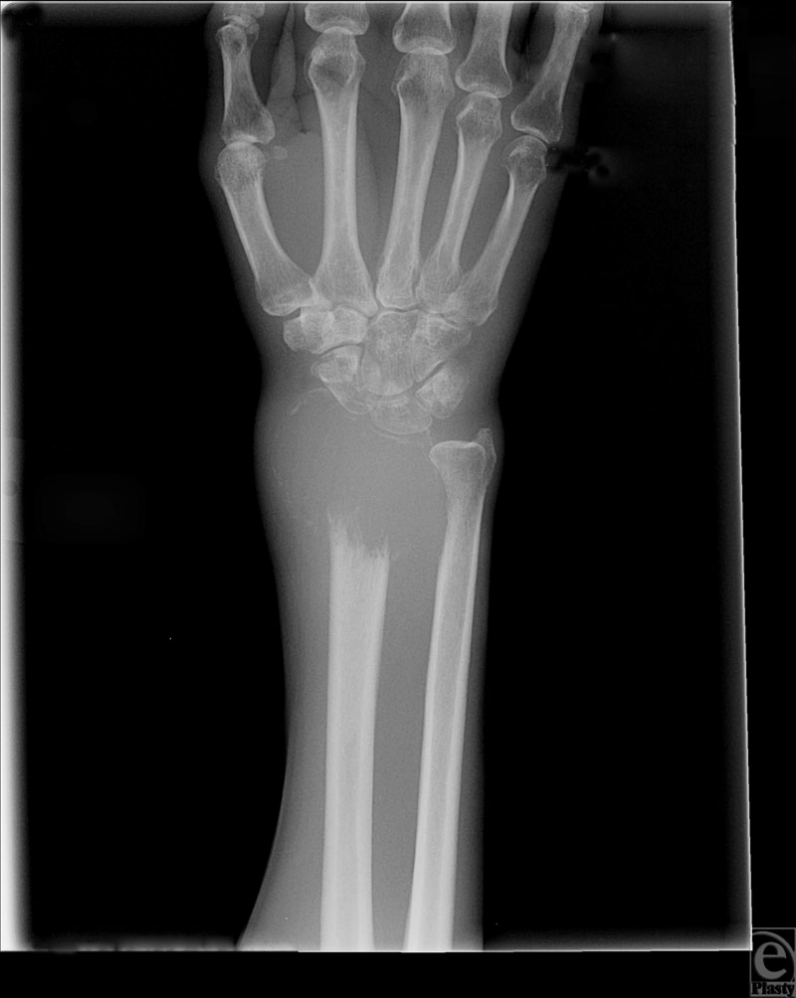
Plain radiograph of Giant Cell Tumor of the Wrist demonstrating an osteolytic lesion of the distal radius.

A plain radiograph of the wrist revealed an osteolytic lesion of the distal radius (Fig 1). Subsequent computed tomographic scan of the wrist exposed an osteolytic lesion measuring 4.6 × 3.9 × 3.9 cm, in the distal radius that extended into the joint with expansion creating a mass effect into the extensor and flexor tendons. Radiographic diagnosis of GCT was confirmed by bone biopsy.

## OPERATIVE METHOD

Tumor excision required en bloc resection that included the original biopsy scar, as well as extensor carpi radialis brevis and longus. Flexor and extensor tendons of the fingers and extensor tendons of the thumb were successfully separated and spared along with the radial artery and vein.

Following en bloc resection of the tumor, pathology revealed involvement of the scaphoid, lunate, and the marrow of the radius, which resulted in further resection. Proximal row carpectomy (PRC) was necessary to achieve clear margins.

The fibula was harvested with the proximal fibular head intact. The fibular collateral ligament was identified and a *z*-plasty was performed to allow a component of the ligament to remain attached to the fibular head for soft tissue ligament reconstruction in the wrist and the remainder used to stabilize the knee following flap removal.

**Figure 2:**
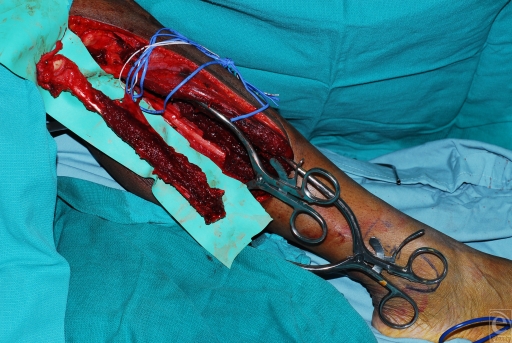
Free fibula graft on peroneal vessels.

**Figure 3:**
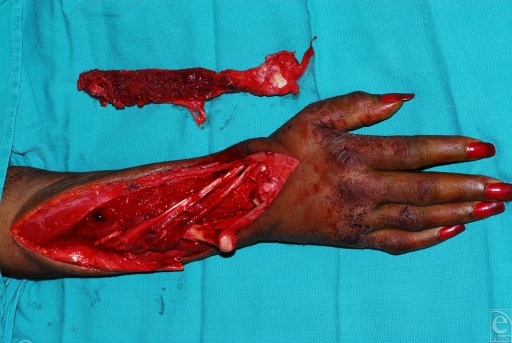
Free fibula graft ready for fixation and vascularization to the radius defect.

The initial fibular flap incision and subsequent dissection were carried down to the peroneus longus and brevis muscles. An osteotomy was performed approximately 19 cm from the head of the fibula. Nutrient vessels were identified entering the fibula at 9, 12, and 15 cm from the fibular head. The fibula flap was harvested on the peroneal vessels (Fig 2). The free fibula flap was transferred to the wrist defect for fixation and the femoral collateral ligament and biceps femoris tendon were anchored to the lateral tibia to maintain knee stability (Fig 3).

**Figure 4:**
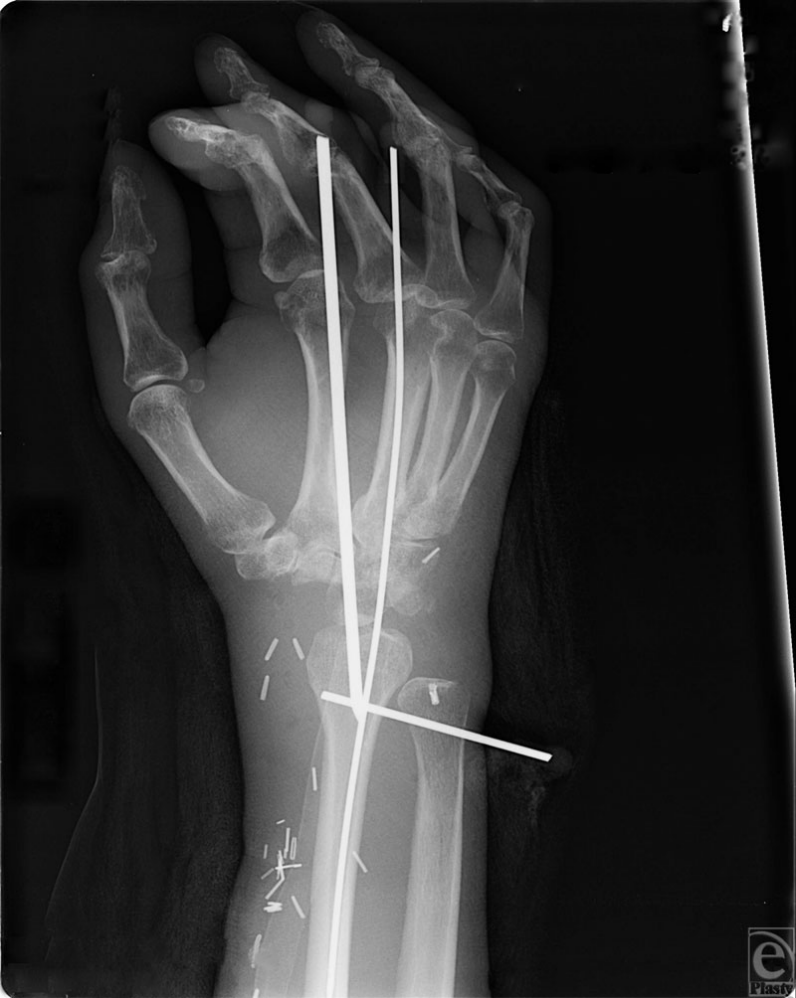
Fixation of the fibula in the wrist with K wires.

Osteosynthesis of the fibula to the radius was performed with a 4-0 compression plate. Additional K wires were placed through the carpus and metacarpals to stabilize the wrist (Fig 4).

Revascularization of the fibula was performed under the surgical microscope with an end-to-side anastomosis of the peroneal artery to the radial artery and an end-to-end anastomosis of the peroneal vena comitante to the radial vena comitante using 9-0 nylon sutures.

The resultant wrist instability of the PRC was addressed with ligament repairs of the radioscaphocapitate ligament. The remnant of the femoral collateral ligament on the fibula head was repaired to the fovea of the ulna. Tendon grafts were used to repair the tendon defects of the extensor carpi radialis longus and brevis left by the resection.

Because of the absence of metastatic disease, the treatment of choice for the patient in this case was en bloc tumor resection with corrective arthroplasty using a vascularized free fibular autograft consisting of the proximal fibular head and 19 cm of the shaft.

The patient tolerated the procedure well and displayed positive findings in follow-up. The patient remained pain-free with no evidence of recurrence. Range of motion was at 50% of preoperative findings leaving her with approximately 35 of wrist extension and 45° of wrist flexion, accompanied by 80% preoperative grip strength (13.1 kg force) as expected after PRC.

## DISCUSSION

Giant cell tumors of bone can present with pain, effusion, swelling, and decreased mobility. In some cases, the initial presentation can be a pathologic fracture. They are commonly found in the epiphysis of long bones with only about 10% occurring in the distal radius. A GCT is mainly benign, locally invasive, but less than 2% can metastasize to the lungs. The choice of treatment can affect the recurrence rate, with up to 50% recurrence in some cases. En bloc resection offers the lowest recurrence rate of less than 10%.^1^

Most bone tumors occur in the metaphysis near the end of long bones, but giant cell tumors occur almost exclusively in the epiphysis. Ten percent of these lesions appear in the distal radius, the third most common site after the proximal tibia and distal femur. In less than 2% of cases, the tumor may metastasize to the lungs.^2^

Multiple therapeutic modalities have shown success in the treatment of GCT. The extent in which the neoplasm is encompassing local structures will dictate the choice of treatment. If the lesion has not expanded beyond the cortex of the bone, a simple curettage of the tumor is indicated. However, simple curettage alone is associated with a 40% to 50% chance of local recurrence. Adjuvant treatment of the bone bed with liquid nitrogen and phenol after removal of the lesion will reduce the risk of local recurrence. Another technique involving the use of intralesional curettage followed by packing the defect with methyl methacrylate has gained popularity because of low cost, ease of use, lack of donor-site morbidity, immediate structural stability, elimination of the risk of transmission of disease associated with the use of allografts, and the potential for earlier detection of local recurrence.^3^

One of the main limitations that restrict the use of most therapies is the size of the lesion. In cases of GCT in the distal radius, a lower rate of recurrence has been reported after the use of surgical resection compared with curettage when the tumor has expanded beyond the cortex. Resection of the lesion will produce a defect in the distal radius requiring reconstruction with either arthroplasty or arthrodesis using vascularized or nonvascularized bone grafts. Bone grafts can be taken from the tibia, the proximal part of the fibula, the iliac crest, or the distal part of ulna in and effort to preserve the motion of the wrist joint.^3^

When considering bone grafts, a fibular transfer is the most suitable technique for a large defect greater than 6 cm in a long bone, because of its length, geometrical shape, and mechanical strength. The use of a vascularized fibula flap offers an effective treatment after ablative resection of locally aggressive benign or low-grade malignant bone tumors. It allows a more radical resection of the pathology without concern for the reconstruction of the resulting massive defect and has the ability to hypertrophy during the healing process.^4^ The use of an autograft is preferred to an allograft for immunologic, infectious, and religious reasons. Under these circumstances, vascularized autogenous fibular bone grafts are commonly used techniques.^5^

Knee instability is a potential complication of harvesting the fibular head for such procedures. In the majority of cases involving fibular free flaps, the proximal head of the fibula is left intact at knee level to maintain stability and the distal fibula is retained to avoid problems with ankle instability.^6^ However, the inclusion of the fibular head in the autograft for carpal reconstruction created instability in the knee that was corrected with anchoring the remnant of the femoral collateral ligament and biceps femoris tendon to the lateral tibia. Potential donor site complications include chronic leg pain, lateral ligament laxity, leg dysesthesia, and foot.

In cases of GCT that have expanded beyond the cortex and require the excision of the distal radius and surrounding structures, the head of the fibula can be included with the free flap for reconstruction of the radiocarpal joint.^6^,^7^ Replacing the distal end of radius with the fibular head to reconstruct the wrist joint can restore function of the carpal joint, which proves to be a safe and effective method with respect to maintaining range of motion in the wrist, preserving grip strength, and minimizing pain.^7^^-^9

Some of the risks and disadvantages involved in GCT resection with arthroplasty using a vascularized fibula free flap include a difficult and lengthy dissection, potential damage to vessels and nerves, donor and recipient site pain and scaring, ischemia to graft and distal structures, weakness, infection, instability of joint, tumor recurrence, and potential for additional surgeries in the event of complications.^3^

## CONCLUSION

The goals of wrist joint reconstruction with a vascularized fibula free flap following en bloc GCT resection include restoration in range of motion and strength, minimizing instability and pain, and preventing recurrence. The literature describes the technique of arthrodesis with a vascularized graft of the fibula shaft as a more useful and reliable procedure for reconstruction of the wrist after excision of the GCT in the distal end of the radius compared to wrist arthroplasty with the vascularized fibular head.^10^^-^14

In the presented case involving GCT excision, success was measured on functionality of the joint, viability of the graft, and the absence of tumor recurrence and pain. The techniques and methods described provide a roadmap for successful excision of a GCT in the distal radius with a PRC and arthroplasty using a vascularized fibula free flap autograft. Based on the outcome of the case, the use of a vascularized fibula free flap following PRC can be utilized in the management of a GCT with a similar presentation.
